# Prevalence of smokeless tobacco use and its impact on periodontal health among adults in Rajnandgaon, Chhattisgarh, India: a cross-sectional study

**DOI:** 10.3389/froh.2025.1659319

**Published:** 2025-09-05

**Authors:** Lubna Tabassum Siddiqui, Sunaina Shetty Yadadi, Anirudh B. Acharya, Marwan Mansoor Mohammed, Saaid Al Shehadat, Vineet Vinay, Vijay Desai, Raghavendra M. Shetty

**Affiliations:** 1 Department of Periodontics, Swargiya Dadasaheb Kalmegh Smruti Dental College and Hospital, Nagpur, India; 2 Department of Restorative Dentistry, College of Dental Medicine, University of Sharjah, Sharjah, United Arab Emirates; 3 Research Institute for Medical and Health Sciences, Microbiota Research Group, University of Sharjah, Sharjah, United Arab Emirates; 4 Department of Oral and Craniofacial Health Sciences, College of Dental Medicine, University of Sharjah, Sharjah, United Arab Emirates; 5 Department of Public Health Dentistry, Sinhgad Dental College and Hospital, Pune, India; 6 Department of Clinical Sciences, College of Dentistry, Ajman University, Ajman, United Arab Emirates; 7 Center of Medical and Bio-allied Health Sciences Research, Ajman University, Ajman, United Arab Emirates; 8 International Adjunct Faculty, Department of Pediatric and Preventive Dentistry, Sharad Pawar Dental College and Hospital, Datta Meghe Institute of Higher Education and Research (Declared as Deemed-to-be University), Wardha, India

**Keywords:** smokeless tobacco, gutkha, periodontitis, clinical attachment loss, gingival recession, rural health, India, SLT

## Abstract

**Background:**

While the detrimental effects of smoking on periodontal health are well-established, the impact of smokeless tobacco (SLT) remains understudied, particularly in rural populations where SLT use is prevalent. The objective of the study is to (1) determine the prevalence of SLT consumption, and (2) evaluate its impact on periodontal health indicators among the adults in Rajnandgaon, Chhattisgarh, India.

**Methods:**

In this cross-sectional study, 1,404 adults from Chhattisgarh, India, were screened, of whom 806 identified as SLT users were further assessed. Demographic data, oral hygiene practices, and SLT consumption patterns were recorded via structured questionnaires. Clinical periodontal parameters, including plaque index (PI), gingival index (GI), clinical attachment loss (CAL), probing pocket depth (PPD), and gingival recession (GR), were assessed. Multivariate regression and structural equation modeling (SEM) were employed to analyze associations between SLT use and periodontal outcomes, adjusting for confounders.

**Results:**

SLT users exhibited significantly worse periodontal health than NTB users, with higher mean CAL (8.7 ± 2.18 mm vs. 3.2 ± 1.45 mm, *p* < 0.001), GR (2.99 ± 1.35 mm vs. 1.05 ± 0.82 mm, *p* < 0.001), and PPD (5.72 ± 1.69 mm vs. 2.91 ± 1.12 mm, *p* < 0.001). Gutkha and Tobacco + Lime demonstrated the strongest associations with periodontal destruction (*β* = 1.82, *p* < 0.01 and *β* = 1.64, *p* < 0.01, respectively). Prolonged SLT use (>10 years), higher frequency (>5 times/day), and lower buccal placement were significant predictors of deterioration (*p* < 0.05). SEM confirmed that SLT type, duration, and poor oral hygiene synergistically exacerbated periodontal damage (CFI = 0.92, RMSEA = 0.04).

**Conclusion:**

The prevalence of smokeless tobacco consumption in Rajnandgaon, Chhattisgarh, was found to be 58.26%, with a higher proportion of users among males (60%) compared to females (40%). SLT, particularly Gutkha, is a significant risk factor for periodontal disease, with usage patterns significantly influencing disease severity. These findings underscore the urgent need for region-specific public health interventions that target smoking cessation and improved oral hygiene practices. Future longitudinal studies should investigate causal mechanisms and the efficacy of interventions.

## 1 Introduction

Periodontal diseases are intricate immunoinflammatory conditions that impact the vital tissues supporting the teeth, including the gingiva, alveolar bone, cementum, and periodontal ligament (1). These disorders primarily arise from the build-up of specific microbiota within the oral biofilm, which engages in a complex interaction with the host's immune system, triggering and perpetuating a chronic inflammatory response. While the microorganisms present in dental plaque serve as the main instigators of periodontal diseases, a range of risk factors can influence the host's immune response to this microbial assault, thereby playing a significant role in both the onset and progression of periodontitis (1, 2).

Tobacco smoking is a substantial risk factor for periodontal disease, causing harmful effects leading to increased plaque accumulation, deeper periodontal pockets, and significant bone loss around the teeth (3, 4). Moreover, smoking compromises the immune response by disrupting the homeostasis of the oral microbiota by fostering the growth of periodontal pathogens. Additionally, the reduction of blood flow and oxygen supply to the gingival tissues hinders the healing processes and regeneration, ultimately worsening oral health outcomes (3–6).

Smokeless tobacco (SLT) encompasses a range of products containing tobacco that are used orally by chewing, placing in the mouth, or sniffing rather than being burned or inhaled (7). While a significant portion of the existing research on tobacco's influence on oral health has predominantly focused on smoking, the harmful effects of SLT products are gaining increasing recognition, particularly in relation to periodontal diseases. SLT exists in various forms, including snuff, chewing tobacco, and betel quid, and is widely used in numerous regions around the world (8).

SLT is used by over 300 million individuals globally and represents a major public health concern. It is responsible for an estimated 4.7 million disability-adjusted life years and contributes to more than 650,000 deaths each year (8–10).

The prevalence of SLT consumption varies widely across regions, reflecting cultural, socioeconomic, and regulatory differences. The global prevalence of smokeless tobacco use shows substantial variation, ranging from 0.1% to 62.2%, based on an analysis of data from 127 countries (8).

As awareness of the adverse health consequences of the SLT products expands, it becomes essential to examine their impact on oral health more closely. Tobacco comprises a complex blend of chemicals, including nicotine, alkaloids, and a host of carcinogens, which can instigate a range of adverse oral health outcomes. Users may experience a spectrum of oral manifestations, including painful oral mucosal lesions, significant gingival inflammation, and alarming periodontal bone loss, all of which contribute to the deterioration of oral health (11).

The relationship between smokeless tobacco consumption and periodontal attachment loss remains somewhat ambiguous, as the literature reveals conflicting evidence. Numerous studies have highlighted a robust association between the use of smokeless tobacco and adverse periodontal outcomes, such as gingival recession, the development of periodontal pockets, and significant bone resorption. In contrast, other research efforts have not succeeded in establishing a meaningful link between smokeless tobacco use and these detrimental effects, resulting in ongoing debates within the scientific community. Despite some variations in findings, the overwhelming consensus among researchers is that smokeless tobacco products adversely affect oral health. They significantly elevate the risk of developing gingivitis, caries, and oral cancer (11–15).

In India, the consumption of smokeless tobacco is notably high, particularly in rural areas where cultural practices are deeply ingrained. Chewable tobacco products dominate, with areca nut combined with slaked lime, catechu, and gutkha leading the way as widely favored choices. Other significant forms include khaini, tobacco lime, and gudakhu, each offering distinct flavours that reflect the strong local custom (16). This widespread usage underscores the urgent need for awareness about its health implications. The existing literature highlights the prevalence and significant impact of smokeless tobacco on periodontal health; however, a notable gap remains in our understanding of the specific types of smokeless tobacco used and their comprehensive effects on the periodontium. Hence, the study was designed with the objective to determine the prevalence of SLT and to evaluate its association with periodontal health indicators among the adults in Rajnandgaon, Chhattisgarh, India, intending to support targeted public health interventions in high-risk communities.

## 2 Materials and methods

### 2.1 Study design

**Figure 1:**
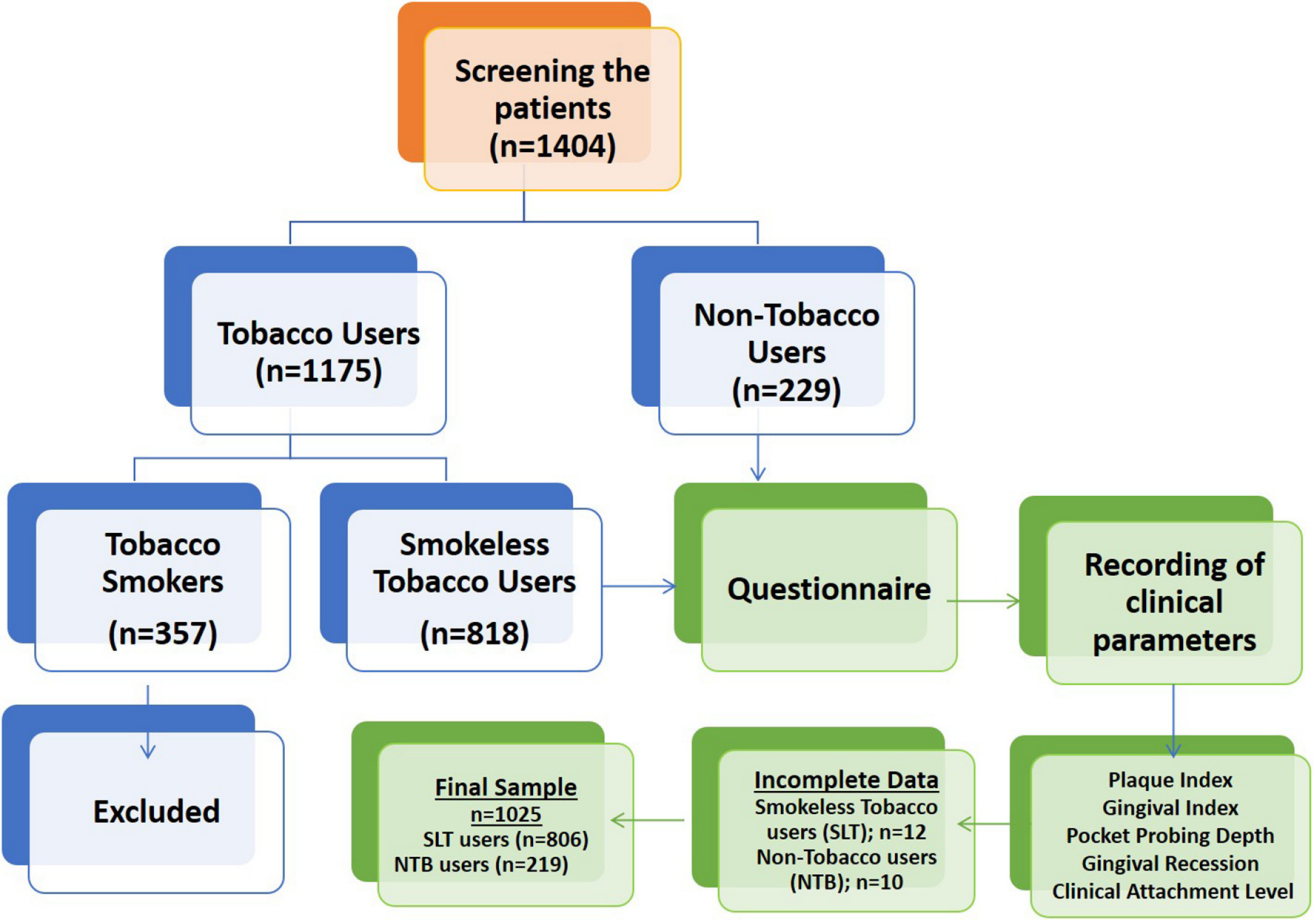
Flowchart of the study.

An observational, cross-sectional study was carried out to assess the prevalence of smokeless tobacco (SLT) use and its effects on gingival and periodontal health in adults attending the Chhattisgarh Dental College and Research Institute (CDCRI), Chhattisgarh, India. This study was conducted in accordance with the 'STROBE” ('Strengthening the Reporting of Observational Studies in Epidemiology”) guidelines (17). The study design is illustrated in Figure 1. SLT users are defined as those who use SLT either daily or occasionally. Non-tobacco users are those who have never used SLT or any type of smoking tobacco.

### 2.2 Research participants and ethical approval

The participants in the study were adults who approached CDCRI for treatment between December 2019 and March 2021. The study was approved by the Institutional Ethical Committee at CDCRI, Rajnandgaon (Ref. No. Perio-16/2019). Before the study began, participants signed written informed consent in either English or Hindi, acknowledging the study procedures and confirming that their involvement was completely voluntary.

### 2.3 Eligibility criteria

The criteria for inclusion consisted of adults aged 18 to 65 years who possess at least two teeth in each quadrant and maintain their dentate status. The criteria for exclusion encompassed individuals with diabetes mellitus, trauma caused by brushing, non-carious cervical lesions, tobacco use, current periodontal treatment, and the utilization of medications that impact periodontal health.

### 2.4 Sample size estimation

The sample size was calculated using OpenEpi's cross-sectional study module, assuming a population of 1,000,000, a hypothesized frequency of 36% based on the Global Adult Tobacco Survey (GATS) Fact Sheet, Chhattisgarh, India (18), 95% confidence level, and a ±5% margin of error. The estimated minimum sample size was 354.

### 2.5 Research information sheet

A structured and validated data collection sheet was utilized to gather socio-demographic details such as age and educational attainment. Information regarding oral hygiene habits—including how often individuals brush their teeth, use dental floss, visit the dentist, rinse their mouths, and use smokeless tobacco (SLT)—was also collected. Furthermore, the questionnaire comprised details about the type of tobacco, daily frequency of SLT usage, duration of SLT consumption each year, placement location of SLT, the duration of SLT placement in the mouth per minute, and the quantity of SLT used (measured in packs).

### 2.6 Clinical examination and clinical parameters recording

The gingival and periodontal conditions of the participants were evaluated using standardized methods, which included the assessment of the Plaque Index (PI), Gingival Index (GI), Pocket Probing Depth (PPD), Gingival Recession (GR) and Clinical Attachment Level (CAL), as outlined below.

The PI was evaluated using a mouth mirror along with a plaque-disclosing solution. Six sites per tooth were evaluated for the presence or absence of supragingival plaque. The plaque was disclosed using a two-tone disclosing solution (Oraldent LTD, UK). The presence (+) or absence (−) of plaque was recorded. Then, the number of positive sites was divided by the total number of sites and presented as a percentage (19, 20). For the GI, the gingiva of each tooth was sectioned into six scoring parts: mesio-facial papilla, facial margin, disto-facial papilla, mesio-lingual papilla, disto-lingual papilla, and mid-lingual margin. Prior to the examination, the gingiva and teeth were gently dried using compressed air or cotton rolls. A periodontal probe was utilized to assess bleeding and inflammation at each scoring unit, employing the following criteria: 0 for no inflammation, 1 for mild inflammation, 2 for moderate inflammation, and 3 for severe inflammation. The GI score for each tooth was determined by aggregating the four recorded scores, and the overall GI score for the individual was calculated by averaging the GI scores from all the examined teeth (21). The probing pocket depth (PPD) was measured using a calibrated color-coded University of North Carolina Probe 15 (UNC-15). The probe was inserted parallel to the tooth's long axis, and measurements were obtained from the bottom of the sulcus or periodontal pocket to the gingival margin at six points per tooth: mesiobuccal, midbuccal, distobuccal, mesiolingual, midlingual, and distolingual (22). Gingival recession (GR) was noted as present when the gingival margin was positioned apical to the cementoenamel junction (CEJ) on either the buccal or lingual surface, and considered absent when the margin was located 1–2 mm coronal to the CEJ (23, 24). Clinical attachment level (CAL) was determined by measuring the distance from the CEJ to the base of the periodontal pocket or sulcus, utilizing a detailed full-mouth periodontal chart (23, 24).

### 2.7 Data collection

Consent was secured from all participants who willingly filled out the questionnaire. Depending on their self-reported use of SLT, participants were classified as either SLT users or NTB. After completing the questionnaire, all individuals who consented underwent clinical evaluations of PI, GI, PDD, GR, and CAL. The quantity of SLT consumed was determined by the number of packs or pieces used, taking into account both the duration and amount, particularly for participants who used various tobacco products. Tobacco chewing duration was assessed through structured interviews using validated questionnaires. All interviews were conducted by a single trained and calibrated interviewer (SSY) using standardized probing questions to minimize bias, and all periodontal examinations were performed by a single trained and calibrated examiner (LS) on the same day. The gathered data was documented and analyzed using statistical methods.

### 2.8 Statistical analysis

The data was collected and entered into Microsoft Excel (version 13) and analysed using IBM SPSS (version 21). For continuous data, mean and standard deviation (SD) were computed, while frequency and percentage were used to summarize categorical data. To compare demographic characteristics and oral hygiene habits between study groups, the Chi-square test was used for categorical data.

For periodontal parameters, an independent t-test was applied to compare PPD, CAL, and GR between smokeless tobacco (SLT) users and non-tobacco (NTB) users. The impact of tobacco placement, duration, and frequency of chewing on periodontal health was assessed using an ANOVA statistical test with *post hoc* Tukey's test. Additionally, a regression analysis was conducted to evaluate the effect of age, gender, brushing habits, and tobacco usage on PPD, GR, and CAL. The Structural Equation Model (SEM) further examined relationships between exogenous and endogenous variables. Statistical significance was set at a *p*-value less than 0.05.

## 3 Results

Among the 1,404 participants screened, 357 (25.42%) were tobacco smokers, 229 (16.31%) were non-tobacco users (NTB), and a total of 818 participants were smokeless tobacco users (SLT), with a prevalence of 58.26%. Smokers were excluded according to the eligibility criteria. A total of 12 SLT users and 10 NTB users had incomplete data and were therefore excluded, yielding 1,025 participants for the final analysis (Figure 1). Among 1,025 participants included in the study, 806 (79%) participants were SLT users with a mean age of 35.54 years, and 219 (21%) were NTB users with a mean age of 26.93 years.

### 3.1 Demographic characteristics and oral hygiene methods across the study groups

Males (60%) were more likely than females to use SLT, whereas females (66%) dominated amongst the NTB users (*p* < 0.001). The SLT users involved 483 (60%) males and 323 (40%) females. The toothbrush was used by the maximum number of participants (86%) in NTB users, compared to only 45% in SLT users. Finger brushing was used as the mode of cleaning by 49.6% of SLT users. The difference in the mode of cleaning between the SLT users and NTB users was statistically significant (*p* < 0.001). However, there was no variation in brushing frequency between the SLT users and NTB users (*p* = 0.46) (Table 1).

**Table 1:** Demographic characteristics and oral hygiene methods across the study groups.

Parameters (*N* = 1,025)	Smokeless tobacco user *n* = 806 (79%)	Non-tobacco user *n* = 219 (21%)	*p*-value^#^
	*n* (%)	*n* (%)	
Gender			
Male	483 (60%)	74 (34%)	<0.001**
Female	323 (40%)	145 (66%)	
Mode of cleaning			
Toothbrush	363 (45%)	188 (86%)	<0.001**
Finger	400 (49.6%)	27 (12%)	
Neem Stick	43 (5.3%)	4 (1.8%)	
Frequency of cleaning			
Once a day	767 (95%)	211 (96%)	0.46
Twice a day	39 (4.8%)	8 (3.7%)	
Mode of brushing			
Horizontal	522 (65%)	52 (24%)	<0.001**
Horizontal and Vertical	284 (35%)	167 (76%)	

# Pearson's Chi-square test.

** Highly Significant.

### 3.2 Type of smokeless tobacco used

The most common SLTs used, categorized by gender, are presented in Table 2. Among the different types of smokeless tobacco used, the most used tobacco products were Gutkha (43%) and Gudakhu (40%) in males, whereas Gudakhu (100%) was used by all the females (*n* = 323) included in the study. The difference in the types of SLT used was statistically highly significant between males and females (X2 = 338.3; *p* < 0.001; Table 2).

**Table 2:** Type of smokeless tobacco used.

Type of SLT	Gender		Significance^#^	
	Males *n* (%)	Females *n* (%)	*X*^2^-value	*p*-value
Khaini	76 (9.43%)	0 (0%)	338.30	0.0001**
Gutkha	350 (43.42%)	0 (0%)		
Tobacco + Lime	57 (7.07%)	0 (0%)		
Gudakhu	323 (40.07%)	323 (100%)		
Total	483 (60.0%)	323 (40.0%)		

# Pearson's Chi-square test.

** Highly Significant.

### 3.3 Comparison of clinical parameters across the study groups

There was a highly significant difference in probing pocket depth (PPD; *t* = 9.69; *p* < 0.001), gingival recession (GR; *t* = 16.16; *p* < 0.001), and clinical attachment loss (CAL; *t* = 13.73; *p* < 0.001) between the SLT users and NTB users (Table 3).

**Table 3:** Comparison of periodontal pocket depth (PPD), clinical attachment loss (CAL) scores, and gingival recession (GR) across the study groups.

Parameters	Smokeless tobacco users (*n* = 806)			Non-tobacco users (*n* = 219)			Significance^#^	
	Mean	SD	SEM	Mean	SD	SEM	*t* value	*p*-value
PPD	5.72	1.691	0.0596	4.57	0.943	0.0637	9.69	<0.001**
CAL	8.7	2.178	0.0475	6.2	1.396	0.0728	13.73	<0.001**
GR	2.99	1.348	0.0767	1.63	1.077	0.0943	16.16	<0.001**

# Independent *t*-test.

** Highly Significant.

### 3.4 Effect of tobacco placement on periodontal health

The placement of tobacco at various sites within the oral cavity considerably influenced periodontal status (*p* < 0.001 for all measurements; Table 4). CAL was maximum among the subjects who inserted tobacco in the lower left buccal and lower right buccal side (9.12 ± 1.229 mm, 9.08 ± 1.30 mm), and they were closely followed by subjects with tobacco placement in all teeth (8.12 ± 2.969 mm). The lowest CAL score (9.00 ± 1.622 mm) was observed among participants who placed tobacco in the lower anterior area. The most severe gingival recession (GR) occurred among participants, placing smoking on the lower left side (3.77 ± 0.962 mm) and right buccal side (3.76 ± 0.957 mm). In contrast, participants using it at any location demonstrated the least GR (1.87 ± 1.026 mm). Probing pocket depth (PPD) was maximum in the group of persons who put tobacco between all the teeth (6.25 ± 2.280 mm), which means greater periodontal damage (Table 4).

**Table 4:** Association of clinical parameters with the location of tobacco placement, duration of tobacco chewing and frequency of tobacco chewing.

Location of tobacco placement	Participants *n* (%)	CAL Score Mean ± SD (mm)	GR Mean ± SD (mm)	PPD Mean ± SD (mm)
Lower Anterior	20 (2.5%)	9.00 ± 1.622	3.10 ± 0.912	5.90 ± 1.07
Lower left buccal	205 (25.4%)	9.12 ± 1.229	3.77 ± 0.962	5.36 ± 0.973
Lower right buccal	258 (32%)	9.08 ± 1.30	3.76 ± 0.957	5.33 ± 0.995
All teeth	323 (40.1%)	8.12 ± 2.969	1.87 ± 1.026	6.25 ± 2.280
Significance	F	10.3	224.3	16.3
	*p*-value	<0.001	<0.001	<0.001
Duration of tobacco chewing	Participants	CAL Score Mean ± SD (mm)	GR Mean ± SD (mm)	PPD Mean ± SD (mm)
0–2 min	241 (29.9%)	7.76 ± 3.174	1.75 ± 1.024	6.01 ± 2.477
2–5 min	82 (10.2%)	9.2 ± 1.902	2.22 ± 0.956	6.98 ± 1.333
5–10 min	33 (4.1%)	9.24 ± 1.3	3.7 ± 0.883	5.55 ± 0.971
>10 min	450 (55.8%)	9.08 ± 1.284	3.74 ± 0.971	5.35 ± 0.995
Significance	F	13.5	228.8	39.7
	*p*-value	<0.001	<0.001	<0.001
Location of tobacco placement	Participants	CAL Score Mean ± SD (mm)	GR Mean ± SD (mm)	PPD Mean ± SD (mm)
1–3 times	144 (17.9%)	7.63 ± 2.98	1.72 ± 1.02	5.92 ± 2.32
3–5 times	297 (36.8%)	8.86 ± 2	3.18 ± 1.32	5.68 ± 1.56
5–10 times	328 (40.7%)	9.02 ± 1.82	3.29 ± 1.2	5.72 ± 1.52
>10 times	37 (4.6%)	8.89 ± 1.47	3.68 ± 1	5.22 ± 1.03
Significance	F	8.86	88.02	3.04
	*p*-value	<0.001	<0.001	<0.001

### 3.5 Effect of tobacco chewing duration on periodontal health

A greater duration of tobacco chewing was correlated with greater periodontal loss (*p* < 0.001 for all parameters; Table 4). The subjects who chewed tobacco for more than 10 min had a high CAL value (9.08 ± 1.284 mm), which was similar to those who chewed for 5–10 min (9.24 ± 1.3 mm) and 2–5 min (9.2 ± 1.902 mm). Conversely, CAL was lowest for those who chewed between 0 and 2 min (7.76 ± 3.174 mm). Gingival recession was higher with longer durations of tobacco chewing, with the maximum GR in the >10 min group (3.74 ± 0.971 mm) and the second highest in the 5–10-minute group (3.7 ± 0.883 mm). Probing pocket depth (PPD) was also similar, with the highest readings in individuals who chewed for 2–5 min (6.98 ± 1.333 mm).

### 3.6 Effect of frequency of tobacco use on periodontal health

The number of tobacco consumption highly affected CAL, GR, and PPD (*p* < 0.001 for all measured parameters; Table 4). The individuals consuming tobacco over 10 times a day showed elevated GR (3.68 ± 1 mm) and PPD (5.22 ± 1.03 mm). Individuals who consumed tobacco between 5 and 10 times a day reported the highest CAL score (9.02 ± 1.82 mm). The minimum CAL and GR were found in people who consumed tobacco 1–3 times a day (7.63 ± 2.98 mm and 1.72 ± 1.02 mm, respectively). Yet, PPD was rather constant between groups, ranging from 5.22 mm to 5.92 mm.

### 3.7 Regression analysis

A regression analysis presents how the various factors relate to the three periodontal indicators: PPD, GR, and CAL. The R-squared (R²) indicates how well the model explains the variance in each dependent variable. R² revealed a variance of 36.8% for PPD, 62.9% for GR (indicating the strongest model fit), and 34.4% for CAL (Table 5a).

**Table 5a:** Regression analysis showing overall model fit between variables and PPD, GR and CAL.

Model	R	*R*²
PPD	0.607	0.368
GR	0.793	0.629
CAL	0.586	0.344

Age was positively correlated with all periodontal measures (PPD, GR, CAL), indicating increased deterioration with age. Males exhibited higher PPD and CAL but lower GR than females. Toothbrush use was associated with better periodontal health compared to finger brushing or neem sticks. Horizontal brushing alone showed greater PPD and CAL than combined horizontal and vertical techniques (Table 5b).

**Table 5b:** Regression analysis showing significant predictors and their effects on PPD, GR and CAL.

PPD					GR				CAL			
Predictor	Estimate	SE	*t*	*p*	Estimate	SE	*t*	*p*	Estimate	SE	*t*	*p*
Intercept^a^	−1.0975	0.9329	−1.176	0.24	1.9659	0.56978	3.45	<.001	0.8683	1.224	0.7094	0.478
Age	0.0764	0.0127	6.003	<.001	0.0251	0.00777	3.235	0.001	0.1015	0.0167	6.0817	<.001
Gender	2.3081	0.3797	6.079	<.001	−0.5922	0.23191	−2.553	0.011	1.7159	0.4982	3.4444	<.001
Mode of cleaning												
Finger—Toothbrush	−0.7292	0.2013	−3.622	<.001	−0.3056	0.12295	−2.485	0.013	−1.0347	0.2641	−3.918	<.001
Neem Stick—Toothbrush	−0.758	0.2403	−3.155	0.002	−0.3024	0.14674	−2.061	0.04	−1.0604	0.3152	−3.3642	<.001
Frequency of cleaning												
Twice a Day—Once a Day	−0.0392	0.2266	−0.173	0.863	−0.0146	0.13841	−0.106	0.916	−0.0538	0.2973	−0.181	0.856
Mode of brushing												
Horizontal and Vertical-Horizontal	−0.3793	0.1762	−2.153	0.032	−0.1477	0.10761	−1.373	0.17	−0.5271	0.2312	−2.28	0.023
Type of tobacco												
Gutkha—Khaini	0.5233	0.1952	2.68	0.008	1.7748	0.11924	14.884	<.001	2.2981	0.2561	8.9718	<.001
Tobacco Lime—Khaini	1.6534	0.2417	6.841	<.001	0.4567	0.14761	3.094	0.002	2.1101	0.3171	6.6549	<.001
Gudhakhu—Khaini	NaN	NaN	NaN	NaN	NaN	NaN	NaN	NaN	NaN	NaN	NaN	NaN
Tobacco chew since (in yrs)												
2–5 Years—0–2 Years	0.4533	0.2602	1.742	0.082	0.3729	0.15895	2.346	0.019	0.8262	0.3414	2.4199	0.016
5–10 Years—0–2 Years	0.2213	0.2568	0.862	0.389	0.2291	0.15685	1.461	0.145	0.4504	0.3369	1.3369	0.182
>10 Years—0–2 Years	0.7091	0.2825	2.511	0.012	0.378	0.17252	2.191	0.029	1.0872	0.3706	2.9337	0.003
Tobacco chew duration (in mins)												
2–5 Min—0–2 Mins	1.0201	0.176	5.797	<.001	0.4721	0.10747	4.393	<.001	1.4922	0.2309	6.4636	<.001
5–10 Min—0–2 Min	0.1179	0.2505	0.471	0.638	−0.0809	0.15298	−0.529	0.597	0.037	0.3286	0.1126	0.91
>10 Mins—0–2 Min	NaN	NaN	NaN	NaN	NaN	NaN	NaN	NaN	NaN	NaN	NaN	NaN
Frequency of tobacco chewing												
3–5 Times—1–3 Times	0.5586	0.1663	3.359	<.001	0.207	0.10158	2.038	0.042	0.7656	0.2182	3.5087	<.001
5–10 Times—1–3 Times	0.545	0.1613	3.379	<.001	0.3625	0.0985	3.68	<.001	0.9074	0.2116	4.2888	<.001
>10 Times—1–3 Times	0.2222	0.2813	0.79	0.43	0.1123	0.17179	0.654	0.513	0.3346	0.369	0.9066	0.365
Location of tobacco placement												
Lower Left Buccal—Lower Anterior	0.2763	0.3416	0.809	0.419	−0.2954	0.20863	−1.416	0.157	−0.0191	0.4482	−0.0426	0.966
Lower Right Buccal—Lower Anterior	0.2102	0.34	0.618	0.537	−0.3711	0.20763	−1.787	0.074	−0.1609	0.446	−0.3608	0.718
All Teeth—Lower Anterior	NaN	NaN	NaN	NaN	NaN	NaN	NaN	NaN	NaN	NaN	NaN	NaN

a Represents reference level.

Linear model contains aliased coefficients (singular fit).

NaN values indicate that the model couldn't compute coefficients due to perfect correlation or lack of variability.

Regression analysis revealed that Gutkha and tobacco plus lime use were significantly associated with increased PPD, GR, and CAL compared to Khaini. A chewing duration exceeding 5 min and a frequency of tobacco usage greater than 5 times per day were significantly associated with higher PPD, GR, and CAL, with lower buccal placement specifically associated with increased CAL (Table 5b).

### 3.8 Structural equation model (SEM) analysis

The outcome of the regression models shows the effect of exogenous variables on the endogenous variables (PPD, GR, and CAL) (Tables 5a, b).

Significant predictors of increased PPD included age (*p* < 0.001), mode of cleaning (*p* = 0.009), brushing technique (*p* = 0.01), type of tobacco used (*p* < 0.001), and duration of tobacco chewing (*p* < 0.001). The results indicate that tobacco chewing habits are strongly associated with deeper periodontal pockets.

GR was significantly associated with the mode of cleaning (*p* < 0.001), type of tobacco (*p* = 0.028), duration (*p* < 0.001), and frequency of tobacco chewing (*p* = 0.001). Tobacco-related variables—particularly type, frequency, and duration—were closely linked to the extent of gingival recession.

Significant predictors of CAL included age (*p* < 0.001), mode of cleaning (*p* < 0.001), type of tobacco used (*p* < 0.001), duration (*p* < 0.001), and frequency of tobacco use (*p* = 0.004). Prolonged and frequent use of smokeless tobacco was associated with greater clinical attachment loss.

## 4 Discussion

This study undertook the estimation of the prevalence of smokeless tobacco (SLT) and a comprehensive examination of the effects of SLT use on periodontal health among adults in Chhattisgarh, India. The prevalence of SLT varies widely due to regional, cultural, and socioeconomic factors. Among adults, SLT use is reported at 29% in India, 26% in Bangladesh, and 22% in Myanmar (8, 25). In India, state-specific data show even higher usage at 35% in Bihar and 33% in Odisha (18). In comparison, rural populations in African countries report lower SLT use, ranging from 5% to 10% (26). A global review of SLT consumption among women of reproductive age noted a wide range of prevalence, from 0.4% to 73%, with the highest rates in Southeast Asia (27). Among adolescents, SLT use has been reported globally at 4.4%, with some rural areas in India showing prevalence as high as 21% (28, 29). In the present study, the prevalence of smokeless tobacco use was 58.26%, with higher usage observed among males (60%) compared to females (40%). This gender disparity aligns with global findings reported by Siddiqi et al. (8), who observed a higher prevalence of smokeless tobacco use among males across 95 countries.

The upper age limit of 65 years was chosen to minimize the confounding effects, such as many individuals exhibit advanced periodontal tissue breakdown independent of tobacco use due to age-related physiological changes (e.g., reduced regenerative capacity, cumulative lifetime biofilm exposure) (30, 31). Also, in our pilot data, >90% of active tobacco chewers in the target population were ≤65 years, as older adults often quit or transition to non-chewing forms.

The use of disclosing agents without explorers or probes was chosen to ensure standardized, reproducible, and objective plaque assessment across all tooth surfaces, including interproximal areas. Unlike tactile methods, which are prone to examiner variability, disclosing agents visually highlight plaque accumulation, enabling consistent quantification across subjects (32). This approach aligns with the study's primary aim to evaluate plaque coverage area rather than thickness or consistency, which would require tactile examination. Similar non-invasive plaque assessment techniques have been validated and widely applied in epidemiological research (19).

According to “Global Adult Tobacco Survey (GATS)” data, the prevalence of smokeless tobacco consumption in Chhattisgarh declined from 47.2% in GATS-1 to 36% in GATS-2, yet it continues to exceed the prevalence of smoking in the region (18). While specific epidemiological data for smokeless tobacco use in Rajnandgaon district are lacking, patterns observed across the state suggest considerable usage in smaller towns and villages (1). In contrast, our study revealed a higher prevalence of 58.26%, which may be attributed to the easy accessibility and aggressive local marketing of smokeless tobacco products. These findings highlight the urgent need for focused public health strategies and community-level interventions to address this issue (27).

The findings of our study revealed significant correlations between SLT consumption and detrimental clinical outcomes related to periodontal health. Analyzing a robust and well-defined population of 1,025 individuals, this research provides valuable insights into the expanding body of evidence suggesting that SLT use is a significant factor in the onset and progression of periodontal disease. This issue is particularly pressing in rural areas, where the prevalence of SLT habits is high, and oral hygiene practices are often inadequate, amplifying the risk of periodontal disease severity and progression (33).

Our findings illustrate that users of SLT products experience greater clinical attachment loss (CAL), gingival recession (GR), and probing pocket depth (PPD) compared to non-tobacco users (NTB), with *p*-values < 0.001 across all assessed metrics. These results robustly validate the hypothesis that, despite being smokeless, SLT products cause significant damage to periodontal tissues and contribute to a serious health burden. The localized placement of SLT within the oral cavity results in prolonged exposure to a myriad of carcinogenic and cytotoxic substances, including nicotine, tobacco-specific nitrosamines (TSNAs), and slaked lime. Such prolonged exposure undermines epithelial integrity, triggers inflammatory reactions, and accelerates the degradation of connective tissue (34). Moreover, users of Gutkha and tobacco-lime demonstrated markedly worse periodontal disease outcomes compared to those who use Khaini. This disparity persisted even after rigorous adjustments for potential confounding factors such as age, gender, oral hygiene habits, and frequency of use. These results align with previous research (14–16, 35), which has underscored the harmful composition of Gutkha, a concoction that contains not only tobacco but also areca nut and slaked lime, intensifying its corrosive potential. In the present study, the regression analysis revealed that Gutkha use is associated with a significant increase in CAL, exceeding 2.29 mm (*p* < 0.001), thereby highlighting its detrimental impact on periodontal health.Our results expand on previous research by providing detailed data regarding the impact of smokeless tobacco (SLT) placement, chewing duration, and frequency, which have often been overlooked. Participants who regularly placed SLT in the lower buccal vestibule showed the highest values for clinical attachment loss (CAL) and gingival recession (GR). This aligns with the findings of Robertson et al. (11) and Chu et al. (36), which emphasized the localized nature of tissue destruction caused by SLT. Furthermore, chewing tobacco for longer than five minutes or more than five times daily significantly worsened periodontal health, indicating a clear dose-response relationship. These findings provide strong clinical evidence that both the intensity and pattern of SLT use modulate periodontal risk.

Age and gender were also found to influence periodontal status, with older individuals and males demonstrating higher CAL and PPD. This is biologically plausible given the cumulative exposure to tobacco and reduced tissue resilience with age. Furthermore, males may exhibit different behavioral patterns related to SLT consumption and oral hygiene that could exacerbate disease progression. Poor oral hygiene practices, especially the use of fingers or neem sticks for brushing, and horizontal brushing techniques, were significantly associated with worse periodontal outcomes, likely due to inadequate plaque removal and soft tissue trauma (12, 16, 37–40).

Our regression and structural equation models (SEM) further reinforced these associations, showing that demographic factors, tobacco type, duration, and frequency of use, and brushing methods collectively explained over 60% of the variance in gingival recession and nearly 37% of the variance in probing depth, demonstrating a strong explanatory power. These quantitative insights are critical, as they suggest that targeted modifications in behavior—such as reducing chewing frequency and improving oral hygiene—could substantially mitigate the risk of periodontitis, even in high-risk populations (13, 34).

The findings from our study are in contrast to those of Bhandarkar et al. (41), who suggested that certain SLT products, such as Khaini and Gutkha, had minimal impact on periodontal health. Such discrepancies may reflect differences in SLT formulations across regions, study design, sample sizes, or the depth of periodontal assessment (41). Notably, our study utilized multiple validated clinical indicators (CAL, PPD, GR) and statistical modeling, which provides a more comprehensive picture of periodontal tissue deterioration.

The biological mechanisms underlying SLT-related periodontal damage are multifactorial. Nicotine absorbed through the oral mucosa disrupts neutrophil chemotaxis, impairs phagocytic activity, reduces fibroblast function, and limits revascularization and wound healing, all of which contribute to progressive attachment loss (42–45). Additionally, local vasoconstriction induced by nicotine may mask early signs of inflammation, potentially delaying diagnosis and treatment. The presence of slaked lime in many SLT products further elevates pH, increasing the bioavailability of free-base nicotine and exacerbating tissue toxicity (46, 47).

This study also highlights critical public health implications. The high prevalence of SLT consumption among males in the rural population, coupled with poor oral hygiene practices and low educational attainment, underscores the need for integrated community-based interventions. Oral health promotion campaigns should be culturally tailored and emphasize not only the risks of SLT consumption but also the importance of effective oral hygiene, including the use of toothbrushes and appropriate brushing techniques.

### 4.1 Strengths, limitations, and future scope

One of the strengths of this research is its stratified analysis of SLT types, duration, placement, and frequency, making it one of the few studies in the Indian context to provide such detailed insights. Moreover, by combining cross-sectional epidemiology with multivariate and structural equation modeling (SEM) analyses, we offer a robust framework for understanding the interplay between behavioral risk factors and periodontal outcomes.

Nevertheless, certain limitations must be acknowledged. Being a cross-sectional study, causality cannot be definitively established. Additionally, self-reported tobacco use may be subject to recall bias or underreporting due to social desirability. The study also did not explore microbiological or immunological markers, which could have helped to elucidate the mechanistic pathways linking SLT use with periodontal destruction. Furthermore, Gudakhu use was exclusive to female participants in this cohort, limiting gender-based comparisons for this particular product. Although we employed rigorous interview methods to assess tobacco chewing duration, some recall bias may remain inherent to self-reported behavioral data. However, our approach aligns with WHO recommendations for tobacco surveillance and has demonstrated validity in similar oral health studies (48, 49).

Despite its strengths, this study is limited by its observational design. Longitudinal studies are required to determine the temporal relationship between SLT consumption and periodontal tissue destruction. Future investigations should also include microbiological and inflammatory biomarker profiling to better characterize the biological impact of various SLT products. Additionally, intervention studies targeting SLT cessation and improved oral hygiene should be conducted to assess the reversibility of periodontal damage and the effectiveness of public health strategies in high-burden settings. Biochemical verification, such as testing for salivary cotinine, was not conducted; however, this can be challenging to implement in large rural populations. The results pertain specifically to SLT usage (such as Gutkha and Gudakhu) in rural Chhattisgarh and may not apply to urban environments or other types of SLT, like snus or betel quid containing tobacco. Although adjustments were made for major confounding variables (such as age and oral hygiene), other unmeasured factors (like diet, genetics, and stress) could still impact periodontal health. Notably, the influence of passive smoking was not evaluated. The exclusion of older individuals (aged above 65 years) restricts understanding of the effects of SLT on older populations, where multiple health issues and accumulated tobacco exposure might increase periodontal damage. Gudakhu usage was limited to female participants, preventing gender-based comparisons for this particular product. Future research should analyze data by gender-related preferences regarding SLT. Incorporating community-based validation methods, such as interviews with family members, could help address this issue in future research.

## 5 Conclusions

The prevalence of smokeless tobacco consumption in Rajnandgaon, Chhattisgarh, was found to be 58.26%, with a higher proportion of users among males (60%) compared to females (40%). This study demonstrated a clear association between smokeless tobacco consumption, particularly Gutkha, and adverse periodontal outcomes, including increased clinical attachment loss, gingival recession, and probing pocket depth. The type, frequency, duration, and placement of tobacco significantly influenced periodontal disease severity and progression.

Demographic and hygiene-related factors, such as age, gender, and brushing methods, also contributed to disease severity. These findings underscore the need for targeted public health interventions that focus on motivation towards cessation of smokeless tobacco consumption and oral hygiene education, particularly in rural communities. Regulation of SLT consumption requires robust implementation and strict enforcement of the WHO Framework Convention on Tobacco Control (FCTC).

## Data Availability

The original contributions presented in the study are included in the article/Supplementary Material, further inquiries can be directed to the corresponding author.
